# The Role of miR-326-3p in Regulating Differentiation and Thermogenesis Genes in Goat Brown Adipocytes

**DOI:** 10.3390/genes16101209

**Published:** 2025-10-14

**Authors:** Yuehua Zhu, Langda Suo, Tingting Jiang, Xinyi Jiang, Yanyan Xia, Linjie Wang

**Affiliations:** 1 Farm Animal Germplasm Resources and Biotech Breeding Key Laboratory of Sichuan Province, College of Animal Science and Technology, Sichuan Agricultural University, Chengdu 611130, China; zzzyova@163.com (Y.Z.); jiangtingting2021@163.com (T.J.); jiangjiangauo@163.com (X.J.); xiayanyi88@163.com (Y.X.); 2 Key Laboratory of Livestock and Poultry Multi-Omics, Ministry of Agriculture and Rural Affairs, College of Animal Science and Technology, Sichuan Agricultural University, Chengdu 611130, China; 3 Institute of Animal Sciences, Tibet Academy of Agricultural and Animal Husbandry Sciences, Lhasa 850009, China; sonaada10@163.com

**Keywords:** miR-326-3p, brown adipocytes, goat, FGF11

## Abstract

**Background**: Brown adipose tissue (BAT) is indispensable for producing heat and contributes critically to the survival of neonatal mammals. MicroRNAs (miRNAs) are small noncoding RNAs that serve as key post-transcriptional regulators, playing a crucial role in regulating BAT development and thermogenesis. However, the role of miR-326-3p in goat brown adipocytes remains largely unclear. **Methods**: Primary brown adipocytes were isolated from goat perirenal adipose tissue and subjected to gain and loss-of-function assays using miR-326-3p mimics and inhibitors. Lipid accumulation, thermogenic-related genes, and mitochondrial gene expression were quantified by Oil Red O staining and qRT-PCR. Target prediction and dual-luciferase reporter assays were performed to validate direct interaction between miR-326-3p and FGF11. **Results**: Expression profiling demonstrated that miR-326-3p is more enriched in brown adipose tissue (BAT) than in white adipose tissue (WAT), and the expression level gradually decreases with adipocyte differentiation. miR-326-3p overexpression significantly inhibited lipid droplet accumulation and the expression of genes associated with differentiation, thermogenesis, and mitochondria, including *PPARγ*, *FABP4*, *UCP1*, and *PGC1α*, whereas inhibition produced the opposite effect. Bioinformatic prediction and dual-luciferase reporter assays further identified fibroblast growth factor 11 (*FGF11*) as a direct target of miR-326-3p. **Conclusions**: These findings reveal that miR-326-3p negatively regulates the differentiation and expression of thermogenic-related genes of goat brown adipocytes, uncovering a novel miR-326-3p-FGF11 regulatory axis.

## 1. Introduction

In mammals, brown adipose tissue (BAT) is a specialized tissue essential for non-shivering thermogenesis, particularly in newborns [1]. Brown adipocytes are abundant mitochondria that contain uncoupling protein-1 (UCP1) and convert chemical energy into heat to maintain body temperature during cold exposure [2]. When lambs are exposed to cold conditions, ample heat can be immediately generated by BAT to maintain their core body temperature, which in turn improves their survival rate [3]. In both human infants and neonatal lambs, BAT depots are mainly located in the pericardial, supraclavicular, cervical, and perirenal regions, but their abundance and activity decline with age [4,5]. A study revealed that, despite the expression level of brown adipose tissue having increased again after humans entered puberty, it remained lower than the expression level during infancy [6]. We previously reported that perirenal BAT is abundant at birth but undergoes a developmental transformation into white adipose tissue (WAT) within the first month of life in goats [7].

A range of hormonal signals and protein factors affect the development and functionality of brown adipocytes [8]. Previous research has demonstrated that microRNAs (miRNAs) act as key transcriptional regulators and potential biomarkers in BAT differentiation and thermogenesis [9,10]. miRNAs, which are short non-coding RNAs averaging 22 nucleotides in length, function by repressing target mRNAs to regulate brown adipocyte differentiation and thermogenesis [11]. Increasing evidence suggests that miRNAs exert their regulatory effects through specific target genes involved in adipogenesis and mitochondrial metabolism. For instance, miR-133 suppresses brown adipocyte differentiation by targeting PRDM16, a master regulator of brown fat identity [12], whereas miR-193b-365 enhances the capacity of brown adipogenesis by repressing *Runx1t1*, an inhibitor of adipocyte differentiation [13]. Similarly, miR-30 stimulates thermogenesis through direct regulation of RIP140, a transcriptional co-repressor of UCP1 [14]. Recent studies have also linked miR-326 to adipogenesis. miR-326 has been reported to inhibit human adipose-derived stem cell differentiation by targeting C/EBPα [15]. We found that miR-326-3p is enriched in goat BAT and suppresses brown adipocyte formation through targeting TLE3 in our previous study [16]. However, the roles and downstream targets of miR-326-3p in the differentiation and thermogenesis capacity of goat brown adipocytes remain poorly understood.

This research explored the role of miR-326-3p in goat brown adipocytes and found that it serves as a negative regulator of both differentiation and thermogenic-related gene expression. Additionally, we found that miR-326-3p directly targets fibroblast growth factor 11 (FGF11). Taken together, these results reveal a novel regulatory connection between miR-326-3p and FGF11 in goat brown adipocytes, offering new insights into the molecular mechanisms that control thermogenic processes.

## 2. Materials and Methods

### 2.1. Animals

This experiment used 8 female Chuanzhong black goats, which were raised at the breeding center of Sichuan Agriculture University, Ya’an, China. 4 one-day-old (body weight 3.16 ± 0.27 kg) and 4 one-year-old goats (body weight 43.77 ± 3.19 kg) were collected. The goats were sacrificed by carotid artery exsanguination after injection of the animal anesthetic, su-mian-xin (0.1 mL/kg BW). The perirenal adipose tissues were obtained from one-day-old goats (*n* = 4) and one-year-old goats (*n* = 4).

### 2.2. Brown Preadipocyte Isolation and Culture

Goat brown preadipocytes were obtained from perirenal fat depots of 1-day-old female Chuanzhong black goats. The tissues were digested using 0.1% (*w*/*v*) collagenase I at 37 °C for 50 min. The digested mixture was passed through a 40 μm cell strainer and seeded into 12-well plates at 37 °C and 5% CO_2_. The cells were refreshed in DMEM/F-12 containing 10% FBS and 1% penicillin/streptomycin. Once preadipocytes reached confluence, induction of differentiation was initiated using DMEM/F-12 medium containing 10% FBS, 850 nM insulin (MCE), 0.5 mM isobutylmethylxanthine (IBMX), 5 μM dexamethasone, 1 μM rosiglitazone, and 1 nM T3 (Sigma-Aldrich, St. Louis, MO, USA). After two days, the culture medium with differentiation medium, which contains 850 nM insulin, 1 μM rosiglitazone, and 1 nM T3. Mature brown adipocytes were collected on day 8.

### 2.3. Total RNA Extraction and cDNA Synthesis

Total RNA, including small RNAs, was isolated from adipocytes using TRIzol reagent (Invitrogen, Carlsbad, CA, USA). For miRNA analysis, reverse transcription was performed using the Mir-X™ miRNA First-Strand Synthesis Kit (Takara, Tokyo, Japan). RNA samples were treated with a gDNA Wiper (included in the reverse transcription kit) prior to cDNA synthesis. For mRNA analysis, cDNA was synthesized using the HiScript^®^ III 1st Strand cDNA Synthesis Kit (Vazyme, Nanjing, China).

### 2.4. Quantitative Real-Time PCR

qRT-PCR was conducted on a CFX96 Real-Time PCR Detection System (Bio-Rad, Hercules, CA, USA) using ChamQ™ SYBR^®^ qPCR Master Mix (Vazyme, Nanjing, China). Expression levels of differentiation genes, brown adipose tissue marker genes, and mitochondria-related genes were quantified. For normalization, TBP was used as the reference gene, based on our previous validation in goat BAT development [17]. The forward primer for miR-326-3p: 5′-CCTCTGGGCCCTTCCTCCAGC-3′. The universal reverse primer (mRQ 3′ Primer) and reference gene U6 quantitative primers were provided by the Mir-X™ kit. Primer specificity was confirmed by melting peak analysis, and amplification efficiency was verified using standard curve analysis (Figure S1). All reactions were performed in technical triplicate. All primer sequences are listed in Table S1.

### 2.5. miRNA Mimic and Inhibitor Transfection

On day 4 of differentiation, adipocytes were transfected with Lipofectamine™ 3000 reagent (Invitrogen, Carlsbad, CA, USA). Chemically synthesized miR-326-3p mimics and corresponding negative control oligonucleotides (mimic NC) were obtained from Sangon Biotech (Shanghai, China). To inhibit endogenous miR-326-3p, chemically synthesized inhibitors and negative control inhibitors (inhibitor NC) were also obtained from Sangon Biotech (Shanghai, China).

### 2.6. Oil Red O Staining

Brown adipocytes were fixed using 4% paraformaldehyde for 15 min at room temperature (RT). Adipocytes were briefly treated with 60% isopropanol for 2 min to prepare for staining. Subsequently, cells were incubated with Oil Red O working solution at RT for 1 h. Images of lipid droplets were captured under an inverted fluorescence microscope (Olympus, Tokyo, Japan). For quantitative analysis, the dye was eluted with isopropanol, and absorbance was measured at 510 nm using a microplate reader.

### 2.7. Dual Luciferase Assay

The 3′ untranslated region (3′UTR) of goat FGF11 and a mutated version (Mut) were cloned into the pGL3-promoter vector (Promega, Madison, WI, USA). HEK293T cells were co-transfected with 200 ng of pGL3-FGF11-3′UTR (WT or Mut) and 10 μL of miR-326 mimic or mimic-NC using Lipofectamine 3000 (Thermo Fisher Scientific, Waltham, MA, USA). After 48 h, relative luciferase activity was determined by TransDetect^®^ Double-Luciferase Reporter Assay Kit (TransGen Biotech, Beijing, China).

### 2.8. Statistical Analysis

Data are shown as mean ± SEM. Student’s *t*-test was used to evaluate differences between two groups, while one-way ANOVA and Tukey’s post hoc test were used to compare multiple groups. All experiments were conducted with at least four independent biological replicates and technical duplicates. Statistical significance was defined as *p* < 0.05.

## 3. Results

### 3.1. Expression of miR-326-3p in Goat Brown Fat

**Figure 1:**
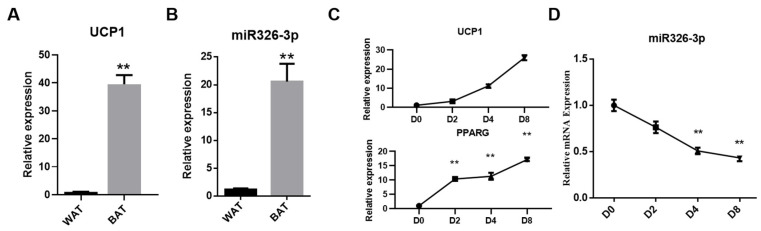
Expression of miR-326-3p in goat brown fat. (**A**): Expression of ***UCP1*** in WAT and BAT. (**B**): Relative expression of miR-326-3p in BAT and WAT (*n* = 4). BAT and WAT samples were obtained from perirenal depots of one-day-old and one-year-old female Chuanzhong black goats. (**C**): Expression of *UCP1* and *PPARG* during brown adipocytes differentiation. (**D**): Expression profile of miR-326-3p at different stages of brown adipocyte differentiation (*n* = 6). Data are shown as mean ± SEM. **, *p* < 0.01.

To verify tissue identity, we first evaluated the thermogenic marker UCP1. As expected, UCP1 expression was markedly higher in BAT than in WAT, confirming the thermogenic phenotype of the BAT sample (Figure 1A). Next, we compared the expression of miR-326-3p between goat BAT and WAT. qPCR analysis revealed that its level was significantly higher in BAT compared with WAT (*p* < 0.01, Figure 1B). Additionally, during the differentiation process, the expression of *UCP1* and *PPARG* gradually increased, indicating successful induction of brown adipocyte maturation (Figure 1C). As primary brown adipocytes differentiated, miR-326-3p expression progressively decreased, with a notable decrease shown on days 4 and 8 in comparison to day 0 (*p* < 0.01, Figure 1B). These results suggest that miR-326-3p is enriched in BAT and dynamically regulated during brown adipocyte differentiation.

### 3.2. Overexpression of miR-326-3p Suppressed the Differentiation and Thermogenic-Related Gene Expression of Goat Brown Adipocytes

**Figure 2:**
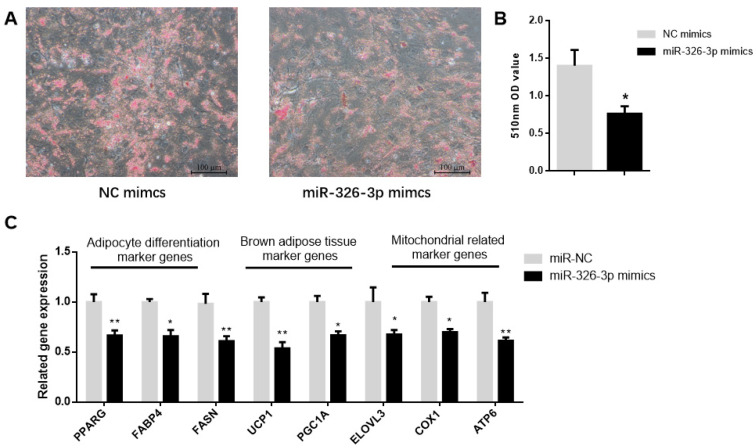
Overexpression of miR-326-3p suppressed the differentiation and thermogenic-related genes of brown adipocytes in goats. (**A**): Oil Red O was applied to brown adipocytes transfected with NC mimics or miR-326-3p. Scale bar = 100 μm. (**B**): Lipid accumulation is measured by OD measurement at 510 nm. (**C**): Relative mRNA expression of differentiation marker genes, BAT marker genes, and mitochondrial-related marker genes, transfection with miR-326-3p mimics (*n* = 6). Data are shown as mean ± SEM. * *p* < 0.05, ** *p* < 0.01.

To determine the function of miR-326-3p, primary brown adipocytes were transfected with either negative control (NC) mimics or miR-326-3p mimics. Oil Red O staining showed a significant (*p* < 0.05) decrease in transfection with miR-326-3p mimic (Figure 2A,B). Consistently, the expression of adipocyte differentiation genes (*PPARγ*, *FASN*, and *FABP4*) as well as brown adipose marker genes (*UCP1*, *PGC1α*, and *ELOVL3*) was significantly (*p* < 0.05) decreased upon miR-326-3p overexpression. Moreover, mitochondrial marker genes (*COX1* and *ATP6*) were also downregulated (Figure 2C). Together, these results indicate that miR-326-3p is involved in the negative regulation of differentiation and thermogenesis in goat brown adipocytes.

### 3.3. Inhibition of miR-326-3p Enhances the Differentiation and Thermogenic-Related Gene Expression of Goat Brown Adipocytes

**Figure 3:**
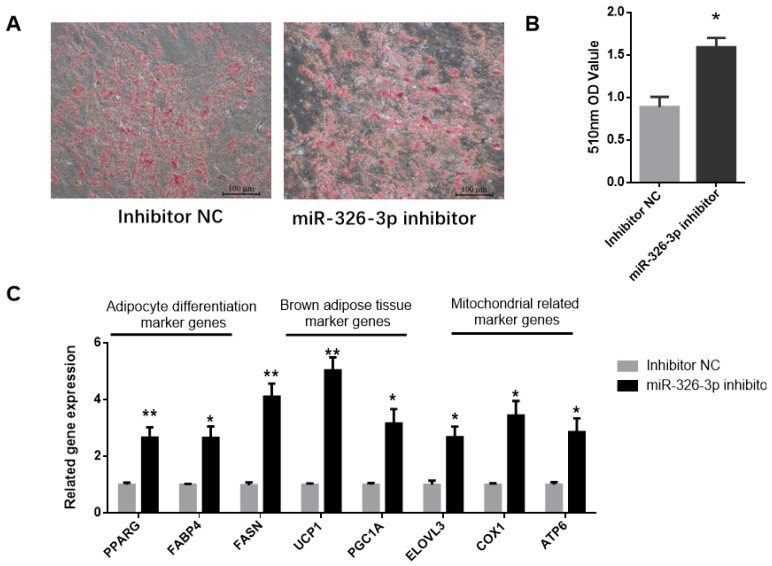
Inhibition of miR-326-3p enhances the differentiation and thermogenic -related genes of goat brown adipocytes. (**A**): Oil Red O was applied to brown adipocytes transfected with NC mimics or miR-326-3p. Scale bar = 100 μm. (**B**): Lipid accumulation is measured by OD measurement at 510 nm. (**C**): Relative mRNA expression of differentiation marker genes, BAT marker genes, and mitochondrial-related marker genes transfection with miR-326-3p inhibition (*n* = 6). Data are shown as mean ± SEM. * *p* < 0.05, ** *p* < 0.01.

To explore the impact of miR-326-3p inhibition, its expression was suppressed in goat brown adipocytes using a specific inhibitor. Oil Red O staining showed that knockdown of miR-326-3p led to a significant (*p* < 0.05) increase in lipid droplet accumulation (Figure 3A,B). Consistently, the expression of adipocyte differentiation genes (*PPARγ*, *FABP4*, *FASN*) was markedly increased (*p* < 0.05, Figure 3C). In addition, inhibition of miR-326-3p significantly (*p* < 0.05) upregulated brown adipocyte marker genes (*UCP1*, *PGC1α*, *ELOVL3*) and mitochondrial genes (*COX2*, *ATP6*, Figure 3C). Together, these findings indicate that reducing miR-326-3p expression promotes the differentiation and thermogenic-related genes of goat brown adipocytes.

### 3.4. miR-326-3p Target the 3′ UTR of FGF11 in Goat Brown Adipocytes

**Figure 4:**
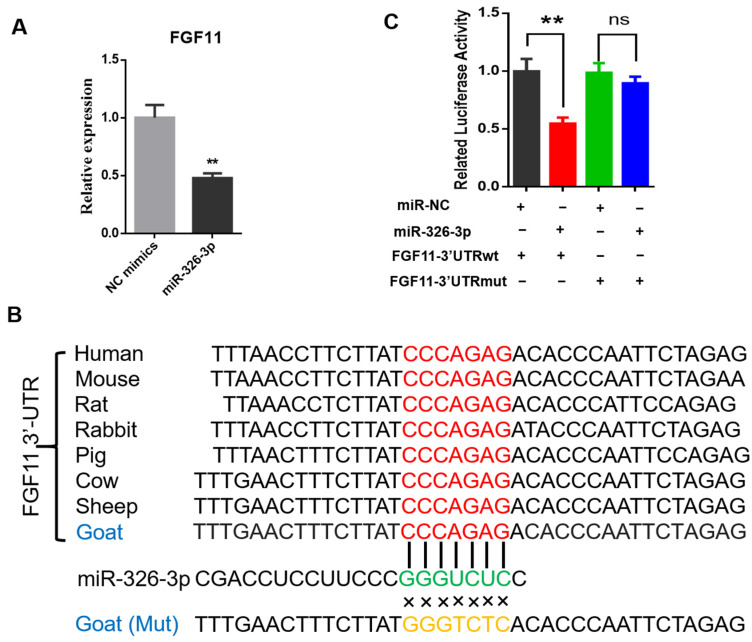
miR-326-3p directly targets the 3′UTR of FGF11 in goat brown adipocytes. (**A**): qPCR analysis of *FGF11* mRNA levels in brown adipocytes transfection with NC mimics or miR-326-3p mimics (*n* = 6). (**B**): Diagram illustrating the predicted miR-326-3p binding region within the *FGF11* 3′UTR across species, together with the designed mutant (Mut) sequence in which the seed-matching bases were substituted. (**C**): Luciferase activity of *FGF11* 3′UTR-WT and 3′UTR-Mut was assessed using a dual-luciferase reporter assay with co-transfection of NC mimics or miR-326-3p mimics (*n* = 6). Data are shown as mean ± SEM. ** *p* < 0.01.

To investigate whether miR-326-3p directly regulates fibroblast growth factor 11 (FGF11), we first examined *FGF11* expression after miR-326-3p overexpression. qPCR analysis revealed that *FGF11* mRNA levels were significantly (*p* < 0.05) reduced in adipocytes transfected with the miR-326-3p mimic compared with the negative control (Figure 4A). Bioinformatic analysis revealed that the predicted binding site for miR-326-3p within the 3′UTR of *FGF11* is highly conserved across several species. In addition, the corresponding mutant (MUT) sequence, in which the seed-matching nucleotides were replaced, was designed to disrupt this potential pairing and was used for subsequent reporter assays (Figure 4B). To experimentally verify this regulatory interaction, wild-type (WT) and mutant (Mut) *FGF11* 3′UTR fragments were cloned into a luciferase reporter construct. There was a pronounced decrease in luciferase activity for the WT construct, while the Mut reporter showed no significant response (Figure 4C). These results provide direct evidence that *FGF11* is a direct downstream target of miR-326-3p in goat brown adipocytes.

## 4. Discussion

Thermogenic activity of brown adipose tissue (BAT) is essential for the survival of newborn animals. In lambs, it represents a major cause of early mortality, and the heat produced by BAT under cold stimulation can account for almost half of the total heat generated [18]. The development of adipocytes follows a sequential process, from mesodermal pluripotent stem cells to progenitors, preadipocytes, and finally mature adipocytes, and this process is tightly controlled by transcription factors, signaling pathways, and non-coding RNAs [19,20]. In the present study, miR-326-3p is enriched in BAT and dynamically regulated during adipocyte differentiation, with expression decreasing as brown adipocytes mature. This expression pattern suggests that miR-326-3p negatively regulates brown adipogenesis and thermogenesis. Functionally, miR-326-3p overexpression inhibited lipid droplet accumulation and suppressed differentiation marker genes, BAT marker genes, and mitochondrial relative genes. In contrast, inhibition of miR-326-3p promoted lipid accumulation and markedly elevated the expression of genes associated with differentiation and thermogenesis. These findings indicate that miR-326-3p exerts an inhibitory role in brown adipocyte differentiation and thermogenic-related genes.

In particular, previous studies have found that the miR-30 family is a key regulator of adipocyte differentiation [21]. Furthermore, miR-30 also contributed to regulating mitochondrial damage and improving mitochondrial dysfunction, while miR-30b/c promoted thermogenesis and white adipose tissue browning [14,22]. Given that the miR-30 family positively regulates both adipocyte differentiation and mitochondrial function, miR-326-3p may function as a competing endogenous RNA, interacting with shared circRNAs or binding sites to modulate the activity of miR-30 family members, thereby fine-tuning brown adipocyte differentiation and thermogenesis. Recent reports implicate lncRNAs and circRNAs in modulating adipogenic pathways through sponging of miRNAs. Mir-326-3p competitively binds with Gm28382 to regulate the role of the chCREB pathway in lipid production in alcoholic fatty liver disease [23]. Our previous findings showed that goat brown adipocytes described circZEB1 regulation of brown adipocyte differentiation via miR-326-3p [24].

Mechanistically, we identified FGF11 as a direct target of miR-326-3p. Dual-luciferase reporter assays confirmed binding of miR-326-3p to the 3′UTR of FGF11, resulting in decreased FGF11 expression. FGF11 has been reported to participate in the differentiation process of human adipose-derived stem cells [25], and FGF11 promotes 3T3-L1 differentiation via regulation of PPARγ [26], which is in line with our identification of FGF11 as a direct downstream target of miR-326-3p. Our previous study suggested that FGF11 has been shown to modulate adipocyte differentiation and thermogenesis, which are essential for lipid metabolism and mitochondrial thermogenesis [27]. Sequence data in miRBase indicate that the mature miR-326 seed sequence is highly conserved among several mammals (including rodents and rabbits), raising the possibility that the regulatory axis described here might extend beyond goats. Given the increasing interest in enhancing thermogenic efficiency and cold tolerance in livestock production, the miR-326-3p–FGF11 pathway may hold potential translational value. In summary, this study identifies miR-326-3p as a factor that negatively regulates adipocyte differentiation and thermogenic-related genes in goats. miR-326-3p is enriched in BAT and dynamically regulated during differentiation, and its overexpression suppresses differentiation and thermogenic gene expression, while inhibition promotes these processes. Mechanistically, miR-326-3p directly targets the 3′UTR of FGF11, thereby reducing its expression. These findings reveal a novel miR-326-3p–FGF11 regulatory axis in brown adipocytes and provide a potential strategy for enhancing thermogenesis and neonatal survival in livestock.

## Data Availability

The original contributions presented in this study are included in the article. Further inquiries can be directed to the corresponding author.

## Supplementary Materials

The following supporting information can be downloaded at: https://www.mdpi.com/article/10.3390/genes16101209/s1, Figure S1: Melt peak and standard curve of miR-326-3p; Table S1: Primers used for qPCR.
