# Supplementary material for: The Role of miR-326-3p in Regulating Differentiation and Thermogenesis Genes in Goat Brown Adipocytes
Source: Genes (Basel). 2025 Oct 14;16(10):1209. doi: 10.3390/genes16101209 (PMC12564603; doi:10.3390/genes16101209)
Supplement: Supplementary file 1 [file genes-16-01209-s001.zip › Table S1.pdf]

Table S1. Primers used for qPCR.

Gene symbol	Sequence 5′-3′	Tm(°C)	Size(bp)
ATP6	TTAGGCCTTCTACCCCACTCA<br>GGATTAGTGGTGTGGGCGTT	60	144
COX1	GAGCCCCCGACATAGCATTT<br>GCTCCTGCATGGGCTAGATT	56.9	160
ELOVL3	ATGAGGCCCTTTTTGGAGGAG<br>CACATCCTCAGTGTCCCGAA	61.4	182
UCP1	ATCTCAGCGGGCCTAACAAC<br>CTTTCCAAAGCCCCGTCAAG	61.4	178
PGC1α	CCACAAATGATGACCCTC<br>GGTTTGGCTTGTAGATGTT	59.0	103
PPARG	GTGTCACTCCTGAACGAAAT<br>GGAAATGCTGGAGAAGTCAA	61.4	156
FABP4	ACTGGGATGGGAAATCAACC<br>CCTTGGCTTATGCTCTCTCG	59.0	117
FASN	CTGCTCAGTGGGCTCCTCA<br>TGGCGGTCAGTGGCTATGT	61.4	187
TBP	TCGCCAAGAATAGTGTGCTG<br>CCGTAAGGCATCATTGGACT	61.3	202
