# Supplementary figures and images for: The Role of miR-326-3p in Regulating Differentiation and Thermogenesis Genes in Goat Brown Adipocytes
Source: Genes (Basel). 2025 Oct 14;16(10):1209. doi: 10.3390/genes16101209 (PMC12564603; doi:10.3390/genes16101209)

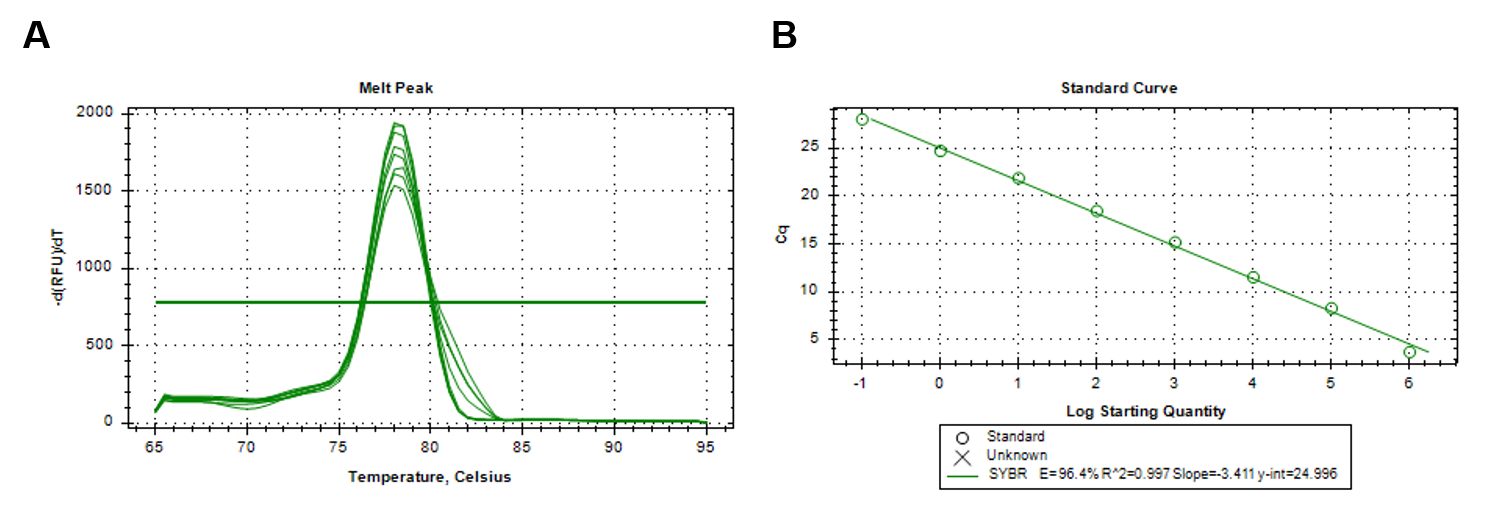

Supplement: Supplementary file 1 [file genes-16-01209-s001.zip › Figure S1.png]
